# Easily Created Prediction Model Using Automated Artificial Intelligence Framework (Prediction One, Sony Network Communications Inc., Tokyo, Japan) for Subarachnoid Hemorrhage Outcomes Treated by Coiling and Delayed Cerebral Ischemia

**DOI:** 10.7759/cureus.15695

**Published:** 2021-06-16

**Authors:** Masahito Katsuki, Shin Kawamura, Akihito Koh

**Affiliations:** 1 Department of Neurosurgery, Iwaki City Medical Center, Iwaki, JPN; 2 Department of Neurosurgery, Itoigawa General Hospital, Itoigawa, JPN

**Keywords:** automated artificial intelligence (autoai), deep learning (dl), delayed cerebral ischemia (dci), machine learning (ml), subarachnoid hemorrhage (sah)

## Abstract

Introduction

Reliable prediction models of subarachnoid hemorrhage (SAH) outcomes and delayed cerebral ischemia (DCI) are needed to decide the treatment strategy. Automated artificial intelligence (AutoAI) is attractive, but there are few reports on AutoAI-based models for SAH functional outcomes and DCI. We herein made models using an AutoAI framework, Prediction One (Sony Network Communications Inc., Tokyo, Japan), and compared it to other previous statistical prediction scores.

Methods

We used an open dataset of 298 SAH patients, who were with non-severe neurological grade and treated by coiling. Modified Rankin Scale 0-3 at six months was defined as a favorable functional outcome and DCI occurrence as another outcome. We randomly divided them into a 248-patient training dataset and a 50-patient test dataset. Prediction One made the model using training dataset with 5-fold cross-validation. We evaluated the model using the test dataset and compared the area under the curves (AUCs) of the created models. Those of the modified SAFIRE score and the Fisher computed tomography (CT) scale to predict the outcomes.

Results

The AUCs of the AutoAI-based models for functional outcome in the training and test dataset were 0.994 and 0.801, and those for the DCI occurrence were 0.969 and 0.650. AUCs for functional outcome calculated using modified SAFIRE score were 0.844 and 0.892. Those for the DCI occurrence calculated using the Fisher CT scale were 0.577 and 0.544.

Conclusions

We easily and quickly made AutoAI-based prediction models. The models’ AUCs were not inferior to the previous prediction models despite the easiness.

## Introduction

A reliable prediction model of subarachnoid hemorrhage (SAH) patient outcomes is needed to decide the treatment strategies and save limited medical resources. Previously, many studies statistically tried to make a prediction model for SAH outcomes [1-8] using neurological severity, age, aneurysm location and size, etc. Their area under the curves (AUCs) of the receiver operating curve varied from 0.70 to 0.90. Gradually, artificial intelligence (AI) has been used to predict the SAH outcomes. Prediction models using random forests had an accuracy of 71% from 147 patients [9] or AUC of 0.84 from 441 patients [10]. Recently, deep learning is being used, and it produces better prediction models with an AUC around 0.90, even with a small dataset [11,12].

Similarly, predicting delayed cerebral ischemia (DCI) occurrence is essential. If we could know the DCI occurrence, we could treat it more prophylactically and immediately. As the most widely accepted radiological scores, Fisher computed tomography (CT) scale [13] and modified Fisher scale [14] quantify the amount of bleeding to predict DCI occurrence. Other statistically calculated scores [15-17] using additional items have been studied, and the AUCs for DCI occurrence are around 0.7 [18]. AI-based prediction models for DCI occurrence had AUCs of around 0.80 [12,19-22]. However, reports on AI-based prediction models for DCI occurrence remain few.

AI will surpass human wisdom, and the technology is gradually used in the neurosurgical fields [9,10,23-31]. However, the majority of medical staff cannot treat AI technology. This is mainly because of the lack of skilled resources to develop a model and time limitations restricting AI application in clinical practice [24]. Recently, automated AI (AutoAI), so-called “the AI by the AI,” has been developed. It is easy to handle by amateurs and will solve these problems with less effort and time. It automatically produces the prediction model within a few minutes as long as the dataset is provided. We herein produced prediction models using the amateur-friendly AutoAI framework, Prediction One (Sony Network Communications Inc., Tokyo, Japan) [32] with an open dataset of SAH patients treated by coiling [33] and compared the utility of the AutoAI-based model to modified SAFIRE score regarding functional outcome and Fisher CT scale regarding DCI occurrence.

## Materials and methods

Study population

We used 298 aneurysmal SAH patients’ open dataset from Iwaki City Medical Center [33]. All patients were treated by endovascular coiling. The surgical indication and general management of SAH and DCI, described as "symptomatic vasospasm," are described in the original articles [33,34]. The treatment is according to the Japanese Guidelines for the Management of Stroke 2009 [35] and 2015 [36]. Notably, patients classified with Hunt and Kosnik grades I-III were considered eligible to undergo aneurysm treatment, whereas those with Hunt and Kosnik grades IV and V were basically not suitable for such treatment. However, patients younger than 65 years with Hunt and Kosnik grades IV or V were considered as candidates for receiving the treatment because of their relatively young age. Those aged older than 65 years were carefully considered for aneurysm treatment, and we treated them only when the patients’ families wanted them to undergo coiling. Therefore, the surgical indication is strictly limited for those with Hunt and Kosnik grade I-III. Rehabilitation of 150 days as maximum and nutritional support were conducted. Prophylaxis and treatment of complications were also ensured. All patients with SAH who had undergone endovascular coiling received 30 mg fasudil three times a day and dual antiplatelet therapy for a month. Intrathecal infusion of 60000 units urokinase for three days after coiling was usually performed to wash out the SAH. Intrathecal, intravenous infusion of nicardipine or intraarterial infusion of fasudil was performed when necessary for treating symptomatic vasospasm. A ventriculoperitoneal shunt was placed when hydrocephalus was observed.

Clinical variables

We collected 24 variables from the dataset: age, sex, height, weight, Hunt and Kosnik grade, administration of antithrombotic drugs, history of smoking and drinking, hypertension, diabetes mellitus, and dyslipidemia. We also collected albumin, white blood cell count, triglycerides, total cholesterol, low-density lipoprotein cholesterol, glucose, hemoglobin A1c, and potassium levels at admission. As radiological findings, aneurysm location, aneurysm size, Fisher CT scale [13], temporal muscle thickness and area as an indicator of systemic skeletal muscle mass were also collected [34,37-45]. All variables can be known at admission.

Outcomes

We set two outcomes. 1) Functional outcomes were assessed using the modified Rankin Scale (mRS) at six months. mRS 0-3 was defined as a favorable outcome. 2) We defined “symptomatic vasospasm” in the dataset as another outcome, DCI occurrence, which was diagnosed by computed tomography angiography, magnetic resonance imaging, or magnetic resonance angiography with symptoms [33].

Making the prediction model by Prediction One

We used Prediction One (version 2.2) to make the prediction model. We divided the 298 patients randomly into a 248-patient training dataset and a 50-patient test dataset. Prediction One read the 248 patients’ data, automatically adjusted and optimized the variables in a way that is easy to process statistically and mathematically, and select an appropriate algorithm with ensemble learning. The missing values were automatically compensated. Prediction One made the best prediction model by an artificial neural network with 5-folds cross-validation. The details are trade secrets and could not be provided.

We let the Prediction One software make two prediction models using the 248-patient training dataset using all 24 variables described above. One was to predict functional outcomes, and the other was to predict DCI occurrence. The AUC of each model and stronger variables were automatically calculated. Then, we performed tests using the 50-patient test dataset. We calculated the AUCs of the models for training and test datasets.

Functional outcome prediction using modified SAFIRE score

As the third model in this study, we investigated modified SAFIRE scores and evaluated its AUCs using the same 248-patient training and 50-patient test datasets. Original SAFIRE score [8] consists of four items: age, World Federation of Neurosurgical Societies grade assessed after neurological resuscitation (rWFNS grade) [8,46], aneurysm size, and Fisher CT scale. rWFNS grade is used in the original SAFIRE score, but we acquired Hunt and Kosnik grade. Therefore, we used Hunt and Kosnik grade instead of rWFNS grade and named this scoring as a modified SAFIRE score (Table 1). After calculating the modified SAFIRE score, we investigated the association between the functional outcomes and the raw total modified SAFIRE score ranging from 0 to 22. Its AUCs for the functional outcome were calculated, and we compared them with those from Prediction One’s model.

**Table 1:** Modified SAFIRE score mRS; modified Rankin Scale, rWFNS; World Federation of Neurosurgical Societies grade assessed after neurological resuscitation, y.o.; years old, *; In this study, we did not use this probability but the total modified SAFIRE score. We evaluate the association of the functional outcomes and the total modified SAFIRE score ranging from 0 to 22. †; Original SAFIRE score uses rWFNS grade. We modified the score using Hunt and Kosnik grade instead of rWFNS grade.

Variables	Points
Size of the aneurysm	
＜10mm	0
10-19.9mm	2
>or =20mm	6
Age	
<50 y.o.	0
50-60 y.o.	1
60-70 y.o.	2
>or 70 y.o.	5
Fisher grade	
1-3	0
4	2
Hunt and Kosnik grade instead of rWFNS grade†	
I	0
II	2
III	3
IV	6
V	9
Total original SAFIRE score [8]*	Risk of mRS 4-6 at 2 months*
0-2	<10%
3-5	10-25%
6-8	25-50%
9-15	50-90%
15-22	>90%

DCI occurrence prediction using Fisher CT scale

As the fourth model in this study, we investigated the relationship between Fisher CT scale [13] and DCI occurrence using the same 248-patient training and 50-patient test datasets. In Japan, original Fisher CT scale is often used instead of modified Fisher CT scale [35,36]. The AUCs for DCI occurrence were calculated, and we compared them with those from Prediction One’s model.

Statistical analysis

The difference between the training and test data was evaluated by Fisher exact test or Mann-Whitney U test using Statcel 4 (OMS Publishing Inc., Saitama, Japan). We calculated AUCs using Python 3.8.5 on Google Collaboratory with NumPy (version 1.19.5), Pandas (1.1.5), Seaborn (0.11.1), and matplotlib (1.3.1).

Ethics

This article uses the anonymized open dataset provided by the other hospital from the Data in Brief journal [33], so we did not need any written informed consent directly from the patients for this article. Therefore, the Itoigawa General Hospital Ethics Committee granted a waiver.

## Results

Clinical characteristics

The 298 SAH patients’ data (mean age 63.7 y.o., 208 women and 90 men) were used, and the details are available in the original article [33]. The mean Hunt and Kosnik grade was 2.62, Fisher CT scale 3.16, modified SAFIRE score 6.20, and the modified Rankin Scale at six months 1.89. Two hundred and eighteen patients (73%) had favorable outcomes, and 57 of the 296 patients (19%) had DCI. The last two patients could not be evaluated regarding DCI due to early death after surgery, so they were removed in the analysis for predicting the DCI occurrence. There were no significant differences in the variables between the training and test datasets.

Model development and test

Prediction One produced each prediction model in less than two minutes, and their AUCs are described in Table 2. The AUCs of the AutoAI-based prediction models for functional outcome in the training and test dataset were 0.994 and 0.801, and those for DCI occurrence were 0.969 and 0.650.

**Table 2:** Prediction models for functional outcome and the occurrence AUC; area under the curve, CT; computed tomography, DCI; delayed cerebral ischemia, *; AUCs were calculated by Python 3.8.5.

Model	AUC* derived from the training dataset (n=248)	AUC* derived from the test dataset (n=50)
Prediction-One-produced model for functional outcome	0.994	0.801
Modified SAFIRE score for functional outcome	0.844	0.892
Prediction-One-produced model for the DCI occurrence	0.969	0.650
Fisher CT scale for the DCI occurrence	0.578	0.544

The stronger variables of each model are listed in Table 3. In the model for the functional outcome, Fisher CT scale, Hunt and Kosnik grade, temporal muscle area, age, white blood cell count were stronger variables in order. In that for DCI occurrence, blood glucose, age, potassium, triglycerides, and albumin were stronger variables in order. Regarding DCI occurrence, Fisher CT scale and Hunt and Kosnik grade were not important variables compared to other variables.

**Table 3:** Stronger variables of each model produced by Prediction One CT; computed tomography, DCI; delayed cerebral ischemia

Variables; order of strength	Model for functional outcome	weight	Model for the DCI occurrence	weight
1	Fisher CT scale	0.121	Blood glucose	0.063
2	Hunt and Kosnik grade	0.108	Age	0.060
3	Temporal muscle area	0.107	Potassium	0.060
4	Age	0.099	Triglycerides	0.060
5	White blood cell count	0.088	Albumin	0.059
6	Height	0.085	Temporal muscle thickness	0.056
7	Temporal muscle thickness	0.083	Temporal muscle area	0.055
8	Aneurysm size	0.082	Aneurysm size	0.052
9	Triglycerides	0.078	Weight	0.050
10	Weight	0.071	Height	0.049
14	Albumin	0.048	Fisher CT scale	0.041
18	Potassium	0.039	Hunt and Kosnik grade	0.026

Comparison to modified SAFIRE score for functional outcome prediction

We calculated the modified SAFIRE score in both datasets. The AUCs of the modified SAFIRE score for the functional outcome were 0.844 in the training dataset and 0.892 in the test dataset (Table 2).

Comparison to Fisher CT scale for the DCI occurrence

The AUCs of the Fisher CT scale for the DCI occurrence were 0.578 in the training dataset and 0.544 in the test dataset. They are inferior to Prediction One’s model (Table 2).

## Discussion

We made two AutoAI-based prediction models, the functional outcome prediction model and the DCI occurrence prediction model. The AUCs of the AutoAI-based prediction models for functional outcome in the training and test dataset were 0.994 and 0.801, and those for DCI occurrence were 0.969 and 0.650. This is one of the few reports using amateur-friendly AutoAI to produce these models. Our study suggested that AutoAI could easily and quickly produce such models in less than two minutes as long as we provide the dataset.

Advantages and limitations of AutoAI

Statistically making a prediction model or scoring system needs a large number of samples over thousands, so these studies tend to be country-initiated or academic association-initiated research with vigorous labor-intensive efforts. However, the larger the sample size, the less detailed information is available, such as comorbidities, use of antithrombotic drugs, or laboratory test data, and the more there are missing data. Also, the treatment strategies vary from hospital to hospital, and patient backgrounds differ depending on countries and regions. Therefore, these prediction models work as the greatest common denominator but not necessarily applicable to the respective hospital.

The performance of our amateur-friendly AutoAI-based model is similar or a bit inferior to these statistically made models. Previous reports on the AutoAI-based functional prediction model for clipped SAH patients [11] and surgically treated intracerebral hemorrhage patients [31] were reported, and they produced good results. Therefore, we attempted to create prediction models similar to the previous reports expecting that we would have got good results with a dataset of patients who were treated by coiling. However, the results were not superior compared to previous scores.

There is a bias in the dataset [33] in our study. The patients were all treated by coiling. Most of the patients with Hunt and Kosnik grade IV and V did not undergo surgery and were rarely included in the dataset. It seems difficult to derive generalizable models from this biased dataset. In the patients treated by the coiling, predicting DCI seems difficult compared to those treated by clipping because the previously reported scores to predict DCI were based on the different sizes and characteristics of cohorts [18]. Regarding predicting outcome, those with Hunt and Kosnik grade IV and V tend to have poor outcomes, so they have strong power to improve the prediction accuracy. Furthermore, there is a difference in the outcome ratios, making it mathematically difficult to make better models. Therefore, our AutoAI-based model using small samples could not conquer the modified SAFIRE score produced using over a thousand samples. Also, our results showed the difficulty of predicting the DCI occurrence based on the information at admission. AutoAI is easy and quick, but it could not make highly accurate prediction models with these small biased datasets. Hyperparameter tuning and data augmentation are ideally needed, but they are difficult for amateur medical personnel.

Recent AI-based prediction models for functional outcome of SAH

Statistical functional prediction scores have been developed [1-8], so AI-based functional outcome prediction aims to surpass these statistical scores. De Jong produced an AI-based prediction model using only four items [12]. Katsuki reported AutoAI based prediction model even with a small dataset of 100 patients, which contributes to decision making according to each hospital’s treatment strategy [11]. Maldaner reported that the AI-based model’s accuracy was improved using the secondary complications and disease information [47]. Including other reports [9,10,48], AI-based prediction for functional outcome has become established. Now AI works on the next stage: predicting rupture risk, automated calculation of hemodynamics, automated morphologic analysis to predict rupture, and automated aneurysm diagnosis [49].

Recent AI-based prediction models for DCI

Ramos used machine learning to make a DCI prediction model using the clinical information, especially radiological features of the aneurysm, and the model had an AUC of 0.74 [20]. Megjhani reported that an hourly risk score for DCI derived from routine vital signs might have the potential to alert clinicians to DCI [21]. De Jong produced an AI-based prediction model using only four items: age, preexisting hypertension, WFNS grade, and modified Fisher scale with an AUC of 0.72 [12]. Park reported an AI-based model with an AUC of 0.77 using many variables, including vital signs and baseline characteristics with minimum redundancy maximum relevance algorithm [22]. Savarraj also reported an AI-based model with an AUC of 0.75 using clinical features [19].

These studies reported that it is now possible to produce AI-based models with AUCs of around 0.75, and that chronological data such as vital signs are important in addition to the patients’ characteristics at admission to increase the accuracies. Our results are inferior to these previous reports, but AutoAI suggests that amateur medical personnel can try to keep up with these cutting-edge researches using an AutoAI.

Limitations of this study

First, we used Hunt and Kosnik grade at admission, but the original SAFIRE score used the WFNS score assessed after neurological resuscitation [8,46] (rWFNS; e.g., cerebral spinal fluid drainage for acute hydrocephalus or evacuation of an intracerebral hematoma). Also, the SAFIRE score predicts two-month outcomes, but our models predict six-month. These are differences, so simply comparing their AUCs requires caution. Second, we used the original Fisher CT scale, which is often used in Japan, but the modified Fisher CT scale [14] is now widely used abroad except for Japan. In addition, other statistically calculated scores [15-17] are also used to predict DCI, so we should compare our model to such recent predicting scores. Third, the prediction model derived from the dataset cannot always be applied to other institutions, and the training and validation dataset must be updated to keep up with advances in medical science and changes in surgical techniques. Fourth, AutoAI produced models easily and quickly, but the neural network architecture by AutoAI is really in the black box for users.

## Conclusions

We easily and quickly made prediction models using the AutoAI framework Prediction One. The accuracies of the prediction models were not so inferior to those of previous statistically calculated prediction models. Even with a small single-center biased dataset, prediction models made by AutoAI might be useful at the institution. AutoAI frameworks are amateur-friendly, so they may be applied to daily clinical practice in the future. The time will come when even amateurs will be able to use AI with ease.
